# Music therapy and Sanfilippo syndrome: an analysis of psychological and physiological variables of three case studies

**DOI:** 10.1186/s13023-021-02123-6

**Published:** 2021-11-20

**Authors:** P. Pérez-Núñez, E. Lázaro, I. Amayra, J. F. López-Paz, P. Caballero, O. Martínez, M. Pérez, S. Berrocoso, M. Al-Rashaida, M. García, A. A. Rodríguez, P. M. Luna

**Affiliations:** Department of Psychology, Faculty of Psychology and Education, University of Deusto, Bilbao, Spain

**Keywords:** Sanfilippo, Music therapy, Psychological and physiological variables

## Abstract

**Introduction:**

Mucopolysaccharidosis type III (MPS III) or Sanfilippo syndrome is a neurodegenerative disease caused by the accumulation of mucopolysaccharides in the body. As the symptoms are wide ranging, it is a challenge to provide a diagnosis and psychological treatment for affected children.

**Method:**

The main objective of this study was to describe a form of music therapy treatment applied to three children diagnosed with MPS III. The psychological variables were evaluated by an ad hoc observation recording template, and the physiological variables were measured with a digital meter before and after each session. The perception of the parents was also considered through a semi-structured interview.

**Results:**

An improvement in the psychological variables was shown in all cases. Changes in the physiological variables were also noted, although they varied according to each child. The parents report some benefit of music therapy and they share difficulty in assessing the extent of benefits of the music therapy.

**Discussion:**

Findings indicate that music therapy can be a useful form of treatment with multiple benefits for children with conditions such as MPS III or similar conditions. However, further research is needed in this area and in the development of specific ways of evaluating music therapy.

## Introduction

Among the approximately 7000 existing rare diseases [1], mucopolysaccharidoses (MPS) are a group of disorders that lie within a heterogeneous group of diseases caused by deficiencies in the lysosomal enzymes, which are necessary to break down mucopolysaccharides, also known as glycosaminoglycans (GAGs). Due to the impaired function of lysosomal enzymes, GAGs accumulate in cells, blood and connective tissue. GAGs are macromolecules that provide structural support to the extracellular matrix and are an important part of cellular regulation and communication processes. Their accumulation results in permanent progressive cell damage that affects many aspects, such as an individual’s physical abilities, mental development and organ function. It is estimated that the collective occurrence of all forms of MPS in newborns ranges from 1 in 10,000 to 1 in 25,000 in Europe [2, 3].

The MPS group is classified into several subgroups, namely: (1) MPS I or Hurler syndrome; (2) MPS II or Hunter syndrome; (3) MPS III or Sanfilippo syndrome (A, B, C and D variants); (4) MPS IV or Morquio syndrome (variants A and B); (5) MPS VI or Marotaux-Lamy; (6); and (7) MPS IX or Natowicz syndrome [4, 5].

MPS III or Sanfilippo syndrome is the most common mucopolysaccharidosis, with an incidence of 0.28–4.1 cases per 100,000 births. The disease is inherited in an autosomal recessive manner and is mainly characterised by severe degeneration of the central nervous system [6, 7]. It was first described in 1963 by paediatrician Sylvestre Sanfilippo, who studied a case of eight children with intellectual disability and elevated urinary excretion of a single GAG, heparan sulphate [8].

The four subtypes of MPS III (A, B, C and D) are classified according to four different enzymatic deficiencies in the heparan sulphate degradation pathway, which are caused by mutations in different genes. The most common subtype is MPS IIIA or IIIB, although this varies between geographical areas [9–11].

MPS IIIA is the best-studied subtype due to its greater frequency, earlier presentation of symptoms and rapid rate of progression [9, 12, 13]. It has recently been suggested that the behavioural phenotype of this subtype has characteristics similar to those described in Klüver-Bucy Syndrome [14]. The progressive loss of language and deficient social interactions characteristic of type A also resemble those that occur in autism spectrum disorders (ASDs) [15]. Patients with MPS IIIB show greater clinical heterogeneity, with either rapid or slow progressors reported even among siblings [16]. While MPS IIIC is less common and MPS IIID is the rarest, both are frequently attenuated forms and few studies are found in this regard [9].

The majority of individuals with MPS III present with a rapidly progressing form of the disease, with the neurological alteration dominating clinically. This is described as being progressive and falls into three stages that start after a period of apparently normal development. In the initial phase, which begins at 1 to 4 years of age, there is a developmental delay, especially in speech and language. The second phase begins at age 3 to 5 years and is characterised by a slowly progressive cognitive deterioration and behavioural alterations, such as impulsivity, hyperactivity, anxiety, sleep disturbances and autistic behaviours. In the third phase, from 10 years old onwards, there is serious dementia and progressive deterioration of the motor function, producing a complete loss of the ability to walk and to swallow. They also have feeding difficulties, seizures, and many patients also develop epilepsy and movement disorders. This loss of abilities often leads to death at the end of the second, or the beginning of the third decade of life [9, 10, 17–21].

Extra-neurological manifestations include recurrent diarrhoea, repeated otorhinolaryngological infections, visual and hearing impairment, coarse facial features, hirsutism, hepatomegaly, dental alterations, cardiac conditions, hernias, upper respiratory infections and musculoskeletal disorders such as osteonecrosis of the femoral head, dysplasia and hip pain, and spine abnormalities such as scoliosis and kyphosis [21–23].

Children with MPS III can easily be misdiagnosed with other pathologies like pervasive developmental disorder, or attention deficit hyperactivity disorder (ADHD) and can be subjected to more invasive tests, dietary restrictions, inappropriate medication or even unproven alternative therapies that are ultimately unnecessary or even harmful [21]. Children with Sanfilippo usually meet criteria for an autism diagnosis, being in risk of having an incomplete diagnosis because the doctor may fail to do a timely and thorough medical workup in order to find the underlying Sanfilippo syndrome. Early diagnosis is essential, since any delay involves a greater burden for family members, in addition to the hardship associated with the disease itself, and all the care tasks to be performed by parents, coupled with the impact this has on their lives [24, 25].

Despite the therapeutic advances, there is currently no cure for this disease, so early diagnosis will help to provide a suitable treatment. Multidisciplinary support treatment is important for this disease because it focuses on ensuring the best possible quality of life and relief from symptoms. This type of treatment covers both physical and psychological aspects. This often involves multiple possible treatments or therapies, such as pharmacological, nutritional support or psychological interventions. Due to the palliative need of these children, since there are currently no treatments to cure the disease, it is very important to consider non-pharmacological alternative therapies, such as music therapy, to improve their quality of life [2, 21].

Music therapy can be used to support patients in different areas, such as prevention, individualized education, rehabilitation, mental health and medical care. This intervention can be targeted at a diverse population since it can be applied both to individuals of all chronological and mental ages, and to individuals with some form of disability or impairment, either physical or mental. There is a need to define the particular effects of music therapy that may help individuals with a range of conditions to improve their well-being, develop creativity, improve learning and interpersonal relationships, manage stress, approach childbirth, and even for pain management and self-fulfilment [26, 27].

While music therapy holds many benefits for treating many diseases, there are no studies on how this intervention can be applied to rare diseases. The theoretical framework and basis used here is the use of music therapy in children with ASD since children with Sanfilippo also develop symptoms of autism. Other disorders that share characteristic symptoms of MPS III, such as ADHD or dementia, have also been examined for the therapeutic use of music therapy. In ADHD, music therapy positively impacts hyperactivity, impulsivity and attention span, while in dementia it has shown benefits in cognitive performance [28–30]. This study considers those physical, cognitive, communicational and emotional aspects on which music therapy can have an effect from a neuroscientific perspective [31].

Calleja-Bautista et al. [32] carried out a review of the literature on music therapy applied to people with ASD in order to analyse its degree of effectiveness. They found that statistically significant improvements had been obtained in 11 out of 18 studies. In the rest, either no statistically significant results had been obtained, or limitations had been found regarding the follow-up evaluations. In the same line, another researcher studied the effects of music therapy in 24 children with ASD and evaluated verbal, non-verbal and social communication by comparing a music therapy group with another group receiving standard treatment. Two blinded evaluators collected the data before and after the interventions and found statistically significant differences in non-verbal communication among participants who had been diagnosed with autism [33]. García [34] conducted a study on music therapy with a 6-year-old boy diagnosed with autism who presented with highly aggressive behaviour within his social environment and total loss of verbal language. Music therapy was used for three months and improvements were shown, as the child began to establish social relationships, control aggressive reactions, and perform simple orders, to which he responded quickly.

An important aspect of music therapy research applied to children with disabilities is the difficulty in collecting data since measuring variables in children with cognitive deficits or an overall developmental delay is a complex process. Therefore, it is essential to have other sources of information such as caregivers’ perception and report. Parents’ views are often considered to assess the influence that music therapy has on their children and family [35–38].

Otherwise, some researches on the use of music therapy are based on physiological data to study if that intervention promotes relaxation or reduces anxiety, stress, pain or cardiovascular problems. Regardless of whether there were changes in the results of these studies, or whether they were significant or not, these physiological indicators, especially heart rate and blood pressure are often used [39–43]. Music therapy was also intended to achieve changes in physiological variables since emotional excitement originates in the autonomic nervous system. Heart rate and blood pressure are commonly used biomarkers that indicate the degree of autonomous activation, which varies due to the influence and interaction between sympathetic activity (involved in stress and activity) and parasympathetic activity (responsible for rest and digestion). The level of excitement is crucial for directing and adjusting emotions and behaviours to be able to adapt. Further, it may be an indicator of changes in stress and anxiety [44, 45].

In sum, scientific literature describes Sanfilippo syndrome as a pathology in which, in addition to physical symptoms, there is a deficit in verbal communication, presence of hyperactivity, impulsiveness, anxiety, sleep disorders and cognitive impairment. Given the lack of adapted assessment and intervention models, it is crucial to develop evidence-based protocols to improve the quality of life of children with this type of pathology, as well as that of their families. On the other hand, music therapy is presented as an appropriate intervention model for this particular pathology due to its clinical profile, although there are hardly any studies that go into detail on the effects of its application [28–34].

In response to these shortcomings, this study aims to describe a music therapy programme applied to children with MPS III (types A and B) and to analyse the psychological and physiological variables related to the symptoms of the disease through quantitative and qualitative observational analysis. This descriptive approach aims to explore this situation to include the music therapy as an intervention and expand the scope of research on rare diseases. This seeks to further the current understanding of the possible benefits of music therapy in children suffering from Sanfilippo.

This paper will answer the following research questions:

- Are clinical changes in physical, communication, social, emotional and cognitive variables identified after music therapy sessions?
- Are there qualitative and quantitative changes in physiological measures (diastolic blood pressure, systolic blood pressure and heart rate) after music therapy sessions?
- Do primary caregivers identify an effect in their child after music therapy sessions?

## Method

### Ethics

This work has been approved by the Research Ethics Committee of the University of Deusto [ETK-3/19–20].

### Participants

Three children aged between 7 and 9 (*M* = 8; *SD* = 1) diagnosed with Sanfilippo syndrome and residing near the study site participated in this study. The participants were two siblings with MPS III type A (a girl and a boy), and a girl diagnosed with MPS III type B. Non-probabilistic, convenience sampling was used as Sanfilippo is a rare disease and the number of patients in a concentrated location is limited. The inclusion criteria to participate in this study was being a child diagnosed with Sanfilippo who attended the Music Therapy Research Centre in Biscay, Spain.

### Instruments

Four different instruments were used for data collection:

1. An ad hoc observation scale was developed to assess the most characteristic psychological aspects of the disease, which were addressed in the music therapy sessions. A template used at Music Therapy Research Centre was considered, as well as scales for people with neurodevelopmental and neurodegenerative diseases who participate in music therapy [46]. Therefore, the assessment sheet presented in “Appendix A” has been developed. A 5-point Likert-type scale was used and the indicators were presented in a negative-to-positive direction depending on the variable in question.
2. *MTS Upper Arm Blood Pressure Monitor 51152 by Medisana* with LCD screen. This is a battery-powered portable computer that uses oscillometric measurement; it is ergonomic and has a compact design, and is suitable for both adults and children. This device measures physiological variables such as systolic blood pressure (SBP), diastolic blood pressure (DBP) and heart rate (HR).
3. An additional instrument used was the ad hoc semi-structured interview. This was applied to the participating children’s parents to discover the children's general clinical history, as well as the differences observed after participating in music therapy sessions. Interviews used in similar studies were considered to create the interview [47–51].
4. The *Barthel Index* was also included [52]. This measured functional independence in personal care and mobility. The Spanish adaptation by Baztán et al. [53] was used, which consists of 10 items that evaluate 10 different everyday activities. Although reliability was not originally determined, Lowen & Anderson [54] obtained *Kappa* indices of between 0.84 and 0.97. In terms of validity, Wade and Hewer [55] obtained correlations between 0.73 and 0.77 with a motor capacity index in a sample of 976 stroke patients.

### Design

This is a study of three cases with mixed methodology. A qualitative descriptive analysis was used by employing an arbitrary observation code and a semi-structured interview. A quantitative analysis was also used by quantifying observation and taking physiological data using the digital meter. Specifically, data were taken in a prospective and longitudinal manner, since the information was collected at different times along three months, and it was a study with an intra-subject repeated measures design.

### Procedure

Children attended 45-min weekly one-to-one sessions at Music Therapy Research Centre in Biscay, Spain. To carry out this study, a total of 20 sessions were observed over three months. All participants obtained permission from their parents through passive consent, after being informed about the study. Before beginning the observational period, the music therapist (MT) provided to the observer the basic sociodemographic data of each case, with the consent of their parents.

The patients went to the Music Therapy Research Centre accompanied by their parents or caregivers. Only the music therapist (MT), the co-therapist (students on the master's degree taught at the centre, who were not always present) and the study's observer were present. The profile of the MT is a pianist who has played in different countries since he was 4 years old, and worked in different fields, including special education and health; he was a university lecturer and held a master's degree in music therapy. The observer was a psychologist specialised in healthcare, with experience with children and adolescents.

The MT welcomed each child when they arrived and took them to the room where the sessions were held, which was located at Music Therapy Research Centre. The room was large and had natural light. It was empty except for an acoustic piano tuned to 432 Hz, a Spanish guitar tuned to 440 Hz, a marimba and a cabinet with instruments in another room. Due to the degree of hyperactivity and impulsivity of the patients, an attempt was made before starting the session to eliminate, cover or block any elements that could hinder the course of the session. There were also some instruments that had been previously selected for each session according to the patient and the pre-established objectives.

The sessions were structured into three parts: The session started with the child listening to the so-called ‘welcome song’ or ‘welcome passage’. This consisted of a few seconds of silence, followed by an improvised passage played by the MT (who was located near and in front of the child) on the classical guitar. The performance was based on the patient’s needs and lasted about two minutes. After this, there were again a few seconds of silence. In this case, the child's role was receptive.

The second part further developed the session by using a more active method, involving vocal or instrumental participation. The patient was invited to sing in an improvised and creative way using their name, the name(s) of siblings, parents, etc. promoting emotional expression, memory and speech. When instruments were used, the patient was offered contact with the instrument (usually piano, guitar, marimba or percussion) and the patient's musical production began. The MT accompanied the sounds made by the child, trying to harmonise with their creation to communicate and encourage integration between child, MT and co-therapist. The rhythms and tones emitted by the patient were not always the same, which required great attention on the part of the MT to adapt to the different pitches and rhythmic context. According to how the patient was seen to respond in terms of their emotions and movement, the therapeutic relationship was aimed at fostering different aspects; for example, percussion instruments were used to work on gross motor control.

The last part of the session used a receptive method, as the first part did. At the end of the session, after asking the patient to take a seat, the MT played a musical passage on the classical guitar for approximately two minutes. Although the passage was improvised, it could be based on the musical passages used during this same session.

Information was collected at the beginning and the end of each session. Both psychological and physiological variables were taken in the room before starting, prior to any musical stimulus. The subsequent measurement was taken at the end of the session, in the same conditions as the initial one, although the psychological measurements were collected within (at the most) 5 min after finishing the session. An audio-visual recording of each session was also made with prior written consent in order to complete the assessment in case the scores were lost.

A semi-structured interview was conducted by the researcher with the parents to expand the information relating to each case, and to gain a qualitative insight into the possible benefits of the therapy. This interview was carried out after completing the observation process three months afterwards, in order to assess the long-term impact of the intervention and to consider the consolidation of the effects of the sessions. The interview was held in a quiet, private place at Music Therapy Research Centre, and it was audio recorded to prevent any loss of information. The parents had previously signed an informed consent form describing the objective of the interview and the study, and indicating that the interview would be recorded. Interviews lasted almost one hour and were performed by the researcher who was a health psychologist.

## Results

### Case 1

#### Clinical and personal history

A. is a 7-year-old boy who suffers from MPS IIIA. He was diagnosed when he was 1 year old, although his symptoms were not recognized until he was 4 years old. There are seven people in the household and he is the youngest of three siblings who all suffer from MPS IIIA. He currently remains in his school year and follows an adapted curriculum. He has a therapeutic teacher that helps him outside the standard school lessons and he also receives speech therapy sessions. According to the *Barthel Index,* he has a moderate dependency. The most notable symptoms are walking on tiptoes and intestinal problems leading to poor sleep quality. He has never been hospitalised and only takes nutritional and vitamin supplements. He has received treatment for deworming, and once a month the mother refers that he goes to energy-based therapies, like energetic kinesiology which focuses on the energetic processes in the body.

#### Analysis of psychological variables in music therapy sessions

##### Researcher's quantitative description

**Fig. 1:**
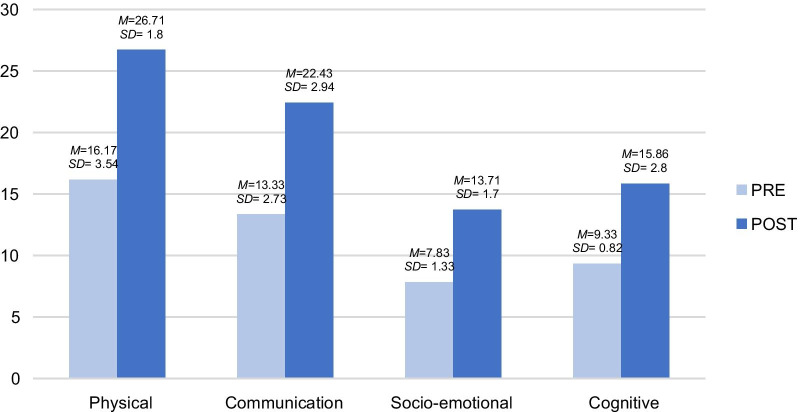
Pre- and post-descriptive analysis of the psychological variables of A. by dimension. The results showed that the total score increased over time in all the dimensions when comparing the pre-treatment measurement to the post-treatment measurement. *M*: Mean *SD*: Standard deviation

This section analyses the calculations related to the variables observed in A.’s seven sessions through the registration template. This was later converted into means and deviations, resulting in the findings shown in Fig. 1. As can be seen, there is a slight clinical improvement in all the post-evaluation carried out, including physical, communication, social, emotional and cognitive variables.

##### Researcher's qualitative description

The researcher’s qualitative observations for the physical, communicative, emotional and cognitive dimensions are described below. A. walked on tiptoes and tried to leave the room and turn off the lights during the sessions. The physical and communication dimensions were particularly remarkable. At the physical level, it was generally difficult to measure his vital signs due to hyperactivity and movements, and it was not possible to carry out measurements in session three. When he listened to the guitar, he moved his legs, although he looked calm (because of his position in the chair), and his breathing was calm and relaxed. However, when he participated actively and played the drum, for example, he did not move his legs. Both verbal and non-verbal communication (sounds) increased when he was stimulated through songs and sounds. At the social-emotional level, positive emotions were strongly expressed and he smiled when he played. Cognitively, his attention was focused on each instrument, and when the MT stopped playing or changed instruments, he also focused on what the MT asked him to do and did himself. He was able to remember as he could repeat his MT’s name and play the instruments; if he could not play a given instrument, he had the capacity to learn. Creative behaviour was observed. For example, if he dropped the drumstick, he continued playing with his hand.

##### Parents’ qualitative description

His mother said that she had seen a great change in her son A. since he had started going to the Music Therapy Research Centre to receive music therapy sessions. At first, he did not like them; he cried and was upset when he had to go; however, she reported that now he is happy and compared with other children suffering from Sanfilippo, she considered that her son’s level of development had improved. For instance, she explained that hyperactivity was less marked and that he smiled a lot and was more obedient, as he paid attention to the instructions received, although she did not attribute it to anything specific. She was happy with her son's general development and said that he was happy when he arrived home after his music therapy sessions.

#### Analysis of physiological variables in music therapy sessions

**Fig. 2:**
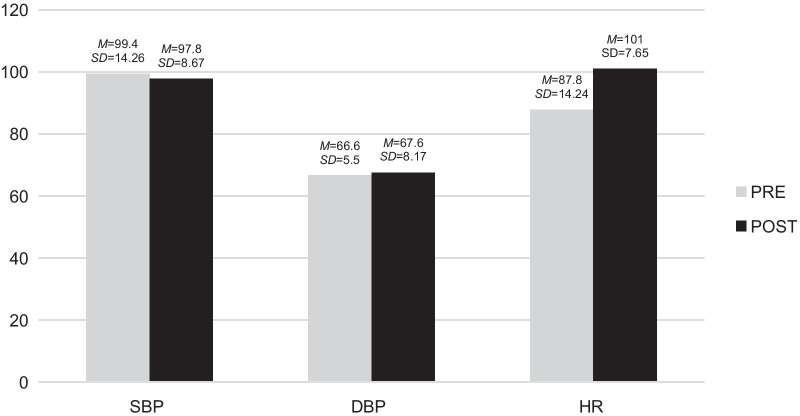
Pre- and post-descriptive analysis of the physiological variables in A.'s case. It was found that his systolic blood pressure (SBP) was higher before participating in music therapy sessions, compared to that measured after the session. However, his diastolic blood pressure (DBP) and heart rate (HR) were lower before starting the sessions, and increased after participating in them. *M*: Mean*. SD*: Standard deviation

These variables were analysed by finding the means and standard deviations of diastolic blood pressure, systolic blood pressure and heart rate that were collected before and after the sessions. The results are shown in Fig. 2.

### Case 2

#### Clinical and personal history

R. is a 9-year-old girl who was diagnosed with MPS III-B when she was 4 years old, although the onset of symptoms was observed at 2 years old, when she stopped talking. She lives with her parents and has had special educational arrangements at school for three years. According to the *Barthel Index,* she has a severe dependency level. The most characteristic symptoms are coarse facial features, hyperactivity and impulsivity. She was hospitalised in 2012 for an operation on her adenoids, and in 2014 to remove her tonsils, as it was causing sleep apnoea. She has septal hypertrophy and had many high fevers and ear infections as a child. She has recurrent respiratory infections, so she takes Flixotide daily, and Ventolin on an occasional basis. She also takes risperidone and diazepam to decrease hyperactivity. She has visited an osteopath and physiotherapist for four years.

#### Analysis of psychological variables in music therapy sessions

##### Researcher's quantitative description

**Fig. 3:**
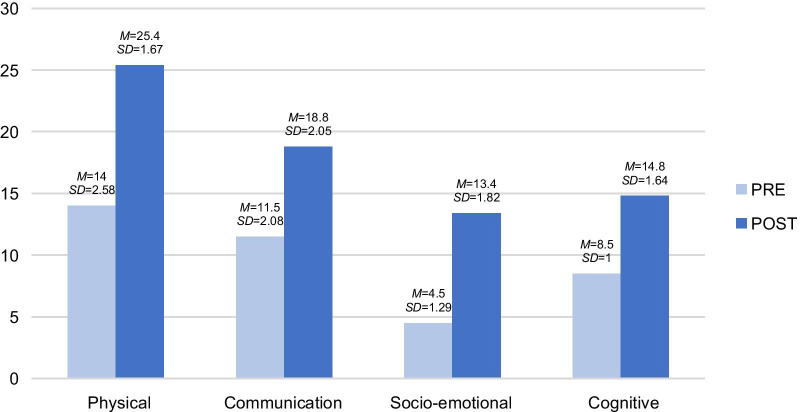
Pre- and post-descriptive analysis of the psychological variables of R. by dimension. The results showed an increase in the total score in all the dimensions, when comparing pre-treatment and the post-treatment measurements. *M*: Mean *SD*: Standard deviation

This section analyses the calculation of the variables observed in the five sessions of R.’s case through the registration template. This was later converted into means and deviations, resulting in the findings shown in Fig. 3. The figure shows a slight clinical improvement in the post-evaluation carried out, including the physical, communication, social, emotional and cognitive variables.

##### Researcher's qualitative description

The researcher’s qualitative observations relating the physical, communicative, emotional and cognitive dimensions are described below. Before starting the sessions, rigidity in the muscle tone of R.’s extremities could be seen. Her breathing was unsteady and she was crying when she arrived. In this case, the most remarkable changes were found at the physical social and emotional level. During the sessions she showed hyperactivity, as she constantly wandered around the room; however, when listening to the guitar, she was able to concentrate on the music being played at that moment and to sit in the chair properly without moving, with slower breathing and a greater level of relaxation. At the communicational level, it is important to highlight her non-verbal responses, as she made a large number of sounds as a response to the MT. The greatest social and emotional change was evidenced by the fact that the crying at the beginning of the sessions disappeared when listening to the guitar. She also became involved with the other people in the room, which fostered group integration. Despite R.’s cognitive deterioration, she paid attention to the music therapist while he played during the sessions.

##### Parents’ qualitative description

The parents highlighted the difficulty in observing changes in their daughter due to the deterioration characteristic of the disease. When she started to attend the sessions, they saw more changes, since they noticed more active participation, more verbal and musical communication, as well as more calmness. Over the past year, they said she had been calm during the session, but when they left, hyperactivity and behaviour problems returned. They also emphasised that it was difficult to see changes due to the subjective nature of the treatment, comparing it with medicine.

#### Analysis of physiological variables in music therapy sessions

**Fig. 4:**
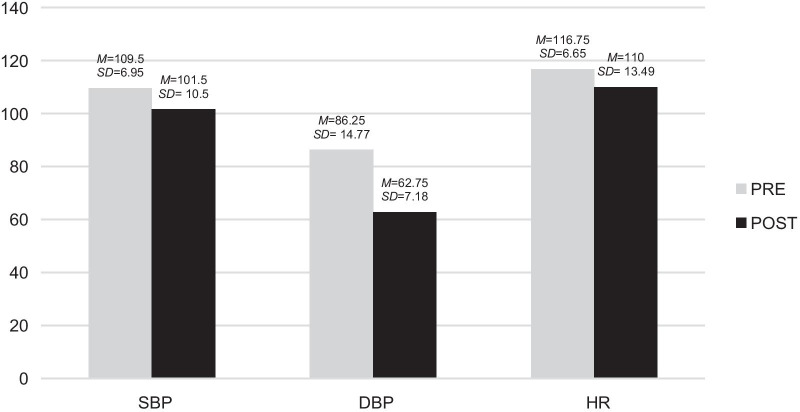
Pre- and post-descriptive analysis of the physiological variables in the case of R. The results showed that all the scores in the physiological variables decreased after participating in the music therapy sessions, the difference in the diastolic blood pressure (DBP) being the most noticeable. *M*: Mean *SD*: Standard deviation

The means and standard deviations of the systolic, diastolic and heart rate presented by R. both before and after the sessions were used to analyse these variables (Fig. 4).

### Case 3

#### Clinical and personal history.

X. is a 9-year-old girl who suffers from MPS IIIA. She was diagnosed when she was 3 years old, at the same age when the symptoms started due to a gait anomaly. There are seven people in the household. She is the middle child, and her two siblings also suffer from MPS III-A. She has special educational arrangements at school and attends speech therapy sessions. According to the *Barthel Index,* she has a severe level of dependency. The most notable symptoms are motor clumsiness and reduced mobility. She had convulsions once and has poor circulation and intestinal disorders that result in poor sleep quality. She also has three crushed vertebrae and scoliosis. She was hospitalised when she was 5 years old to have her adenoids removed due to apneas at night and otitis. She takes natural enzymatic and vitamin supplements. She usually has intestinal parasites and receives treatment for deworming, and the mother mentions that once a month she goes to energy-based therapies like energetic kinesiology which focuses on the energetic processes in the body.

#### Analysis of psychological variables in music therapy sessions

##### Researcher's quantitative description.

**Fig. 5:**
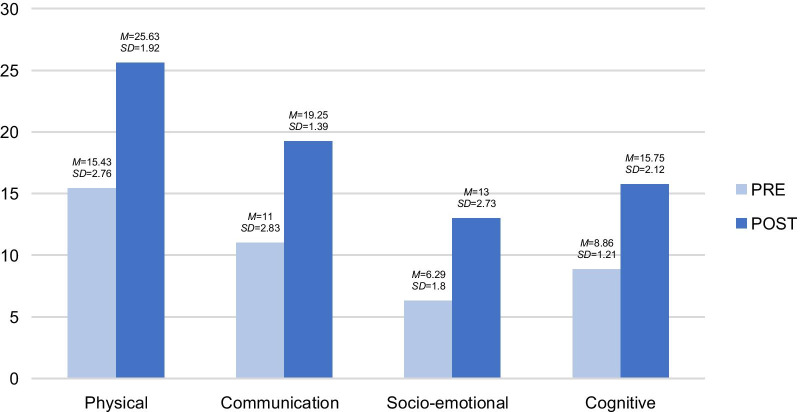
Pre- and post-descriptive analysis of the psychological variables of X. The results showed a general tendency for the total score to increase in all the dimensions, comparing the pre-treatment and post-treatment measurements. *M*: Mean *SD*: Standard deviation

This section analyses the calculation of the variables observed in the eight sessions of X.’s case through the registration template. This was later converted into means and deviations, resulting in the findings shown in Fig. 5. As can be seen in the figure, a slight clinical improvement is obtained in all the post-evaluation carried out, including the physical, communication, social, emotional and cognitive variables.

##### Researcher's qualitative description

The researcher’s qualitative observations relating to the physical, communicative, emotional and cognitive dimensions are described below. Before beginning the sessions, there was restlessness in X.’s legs and strong emotions, notably anguish and general restlessness. Restlessness in her legs diminished with music, and her level of relaxation increased. She communicated by repeating the sounds made by the MT, or creating her own singing, and playing the instrument played by the MT. At the gestural and body level, she frequently held the MT’s hand and maintained visual contact with him. The social and emotional aspects were also intensified, with positive or neutral emotions predominating. X. smiled at the MT and approached the people who were present, showing group integration. At the cognitive level, the most remarkable thing was her ability to pay attention when the MT played an instrument, and her creativity, as she created new harmonies by singing.

##### Parents’ qualitative description

X.’s mother considered the role of music in people's lives to be fundamental. She mentioned the difficulty in terms of being able to observe changes, and of assessing what remained after leaving the sessions, given the neurodegenerative disease suffered by her daughter. X. really likes music, and she sings and dances daily. This is a way of communicating with her that her mother has used since childhood. X.’s mother reported that, in general, X. was relaxed when she went home after the sessions. For example, there were days that she cried when she went to the session due to back pain. However, when she came out of the session, she felt more relaxed. Her mother stated that X. clearly enjoyed the sessions.

#### Analysis of physiological variables in music therapy sessions

**Fig. 6:**
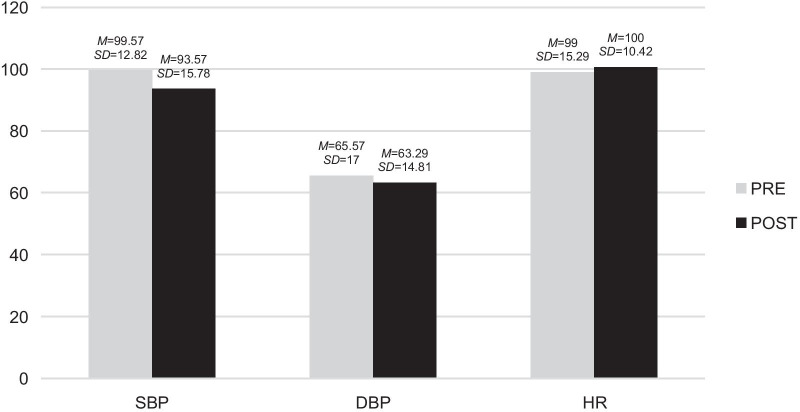
Pre- and post-descriptive analysis of the physiological variables in the case of X. The results showed that both her diastolic blood pressure (DBP) and systolic blood pressure (SBP) decreased after the music therapy session, while her heart rate (HR) increased slightly. *M*: Mean *SD*: Standard deviation

These variables were analysed by finding the means and standard deviations of X.'s diastolic blood pressure, systolic blood pressure and heart rate that were collected before and after the sessions. The results are shown in Fig. 6.

## Discussion

This study uses a mixed methods approach to describe the effects of music therapy on three patients with Sanfilippo syndrome. This was done by measuring which psychological (measured qualitatively) and physiological (measured quantitatively) variables in these children increased or decreased after the music therapy intervention.

There were higher scores after music therapy sessions in the four psychological variables included in the present study in all cases. This showed an improvement in terms of physical, communication, social, emotional and cognitive levels after participating in music therapy sessions. Some variations were found in the dimensions according to each child’s specific needs and limitations, which were shown in the measurements prior to the sessions. In the case of A., the physical and communication dimensions prevailed since the extent of his disability in these areas was less marked than in the other cases. He was the only one that maintained his verbal abilities and used them in the sessions. In the case of R., the changes at the physical level stood out because hyperactivity was particularly marked initially although it decreased considerably during the sessions. Besides, in R.’s case, an improvement was particularly noticeable in the social and emotional aspects, since she cried before starting the sessions and but this decreased as the session progressed. X. experienced improvements especially on a physical level, due to the general restlessness shown before the sessions and the relaxation during the therapy.

These results can be compared with those obtained from children with ASD. García [34] observed changes in different parameters in a child with autism after a music therapy intervention. At a physical or body level, the child’s levels of aggressiveness decreased; at the social and emotional level, she began to establish more social relationships and at the cognitive level, she began to pay attention to instructions. Gattino et al. [33] also found a significant improvement in non-verbal communication among children with autism. This improvement on the psychological level was also observed in the review conducted by De Vries et al. [56] on music therapy in children with autism. They found an increase in attention and concentration, in social response behaviour and verbal communication, greater recognition and/or understanding of emotions and a decrease in anxiety.

On a different note, the assessments made by the parents varied considerably. However, in all cases, the parents stated that, to a greater or lesser extent, they had seen that music therapy had benefited their children, especially at a social-emotional level. Furthermore, the common point of all the cases was the difficulty in making long-term evaluations of music therapy, due to the degenerative character of the disease. This information highlighted the importance of working with families in this type of study. Parents’ views were essential because their involvement provided a source of external feedback for the researcher that went beyond what was observed in the sessions. In other studies, in which parents have been interviewed, in addition to finding positive opinions about the use of music therapy, researchers also stressed the importance of their collaboration [35, 36, 38].

The physiological variables showed greater variability in the results. This could be due to each child’s varying needs and baseline activation level. In the cases in which it tended to increase, the pre-treatment measurement was low whereas, in those where it tended to decrease, the pre-treatment measurement was in the normal average range or slightly above, so it was adjusted to what each individual needed at the time. To a greater or lesser extent, systolic blood pressure decreased in all cases. The heart rate was found to have increased in the siblings, which may have to do with the level of activation that the sessions produced on them, as a way of preparing the body for the action required. In the case of R., the pre-treatment measurement was higher in all measures, which may be due to the fact that her activation level was higher, especially due to crying and hyperactivity. After the sessions, all the indicators decreased to a normal range level, and a balance was achieved between the sympathetic and parasympathetic nervous systems [44, 45].

Although no studies have been conducted on the measurement of physiological variables in children with MPS III or similar diseases when participating in a music therapy treatment, these results can be compared with research carried out on dimensions that are affected in these children, such as pain, stress and anxiety. In these studies, there was a decrease in the relevant indicators, with increased relaxation and reduced pain, anxiety and stress, as in the case of R. and in all cases in systolic blood pressure [39–43].

This study has some limitations that should be considered for future research. Mainly, it should be noted that, since it is a rare disease and it is difficult to put together a sample, the number of the selected sample is small, with only three participants. Given the small sample size, the results were not compared between the cases, as it was considered that the results would not be relevant and would not be an appropriate representation of reality.

Another limitation was the measure used to collect psychological variables. This was an ad hoc template on the direct observation of these variables by the researcher. It could lead to the impression of being subjective judgment, in which the observer’s perspectives and wishes may have influenced observation, although an attempt was made to overcome this limitation by recording the sessions. For future studies, it would be important to introduce different observers and calculate the interjudge agreement as a measurement of reliability. In addition, as it is a non-standardised instrument, despite relying on validated measures, some studies would be needed to determine its reliability and validity.

Continuing with the assessment, another limitation is that due to the degree of affectation of the children and their ages, a standardised evaluation of the variables studied was not carried out. As the sample was small, a qualitative descriptive approach was chosen in order to obtain information of clinical and scientific interest. However, it would be interesting, for future studies and for international comparisons, to use standardised instruments, especially through a standardised parental report.

A final limitation to note is the lack of previous research on the subject, which makes it very difficult to compare or comment on the results found in this study. Although it has been based on research on music therapy and other diseases that have similarities with Sanfilippo, there are no previous studies on this disease in particular or any type of non-medical treatment. However, this type of limitation can serve as an opportunity to identify new gaps in the literature and consequently stimulate new research.

Regarding the implications of this preliminary clinical report, the results could be useful to encourage the development of a music therapy programme not only for children with MPS III but for anyone suffering from a rare disease or a neurodegenerative disorder of this nature that involves verbal and cognitive impairment. It can also influence the creation of a validated test to reliably and validly evaluate music therapy sessions, which could lead to a more quantitative study to determine if the differences are significant. It is also important to highlight the importance of this type of studies where the purpose is to gain a better understanding about the effectiveness of this intervention among this group of patients since it could be applied at an earlier age when the deterioration is not so marked.

## Conclusions

The results of this study provide preliminary information and indicate positive changes in both psychological and physiological variables after having received music therapy sessions. This leads to the conclusion that this treatment is a promising practice for improving quality of life. The study provides information that has not been published to date and can be of great social importance, as it can be very helpful for professionals and family members of those affected. These data could serve as a basis for future research that has greater resources available, such as a larger sample, rigorous design and a standardised assessment and treatment. Also, further research should be done both on this rare disease and music therapy so that more can be learnt about them and to clarify whether this treatment is effective.

## Data Availability

The datasets generated and/or analysed during the current study are not publicly available because they belong to the University of Deusto, but are available from the corresponding author (Paula Pérez Nuñez) on reasonable request.

## Appendix A: Observational scale

**Table:**

	1 LOW	2 MEDIUM–LOW	3 MEDIUM	4 MEDIUM–HIGH	5 HIGH
1. Physical					
Posture					
Muscle tension (relaxed or rigid)					
Breathing					
Gaze					
Visual tracking					
Relaxation					
2. Communication					
Verbal					
Non-verbal (sounds)					
Gestural and body language (hands)					
Facial expression					
Eye contact					
3. Socio-emotional					
Expression of emotions					
Mood					
Group integration					
4. Cognitive					
Attention					
Focusing attention without being distracted					
Memory					
Creativity					
$$\begin{aligned} &{\text{PRE}}\,{\text{SBP}} \to \quad \quad {\text{POST}}\,{\text{SBP}} \to \\ &{\text{PRE}}\,{\text{DBP}} \to \quad \quad {\text{POST}}\,{\text{DBP}} \to \\ &{\text{PRE}}\,{\text{HR}} \to \quad \quad {\text{POST}}\,{\text{HR}} \to \\ \end{aligned}$$					

PATIENT:

SESSION No.:

DATE:

→ **RESEARCHER'S NOTES**

## Abbreviations

MPS: Mucopolysaccharidosis
MPS III: Mucopolysaccharidosis type III
GAG: Glycosaminoglycans
ASDs: Autism Spectrum Disorders
ADHD: Attention Deficit Hyperactivity Disorder
SBP: Systolic Blood Pressure
DBP: Diastolic Blood Pressure
HR: Heart Rate
MT: Music therapist
